# Disparities in prehospital and emergency surgical care among patients with perforated ulcers and a history of mental illness: a nationwide cohort study

**DOI:** 10.1007/s00068-023-02427-1

**Published:** 2024-02-14

**Authors:** Julie Mackenhauer, Erika Frischknecht Christensen, Jan Mainz, Jan Brink Valentin, Nicolai Bang Foss, Peter Olsen Svenningsen, Søren Paaske Johnsen

**Affiliations:** 1 Danish Center for Health Services Research, Department of Clinical Medicine, Aalborg University, Fredrik Bajers Vej 5, 9220 Aalborg Ø, Denmark; 2 Psychiatry, Aalborg University Hospital, 9000 Aalborg, North Denmark Region Denmark; 3 Department of Sociale Medicine, Aalborg University Hospital, Aalborg, Denmark; 4 Centre for Prehospital and Emergency Research, Department of Clinical Medicine, Aalborg University and Aalborg University Hospital, 9000 Aalborg, Denmark; 5 Prehospital Emergency Medical Services North Denmark Region, 9000 Aalborg, Denmark; 6 Department of Community Mental Health, Haifa University, Haifa, Israel; 7 Department of Health Economics, University of Southern Denmark, Odense, Denmark; 8 Department of Anaesthesia and Intensive Care Medicine, Hvidovre Hospital, Institute for Clinical Medicine, University of Copenhagen, 2650 Hvidovre, Denmark; 9 Department of Surgery, North Zealand Hospital, Copenhagen University Hospital, 3400 Hillerød, Denmark

**Keywords:** Mental disorders, Emergency treatment, Peptic ulcer perforation, Healthcare disparities

## Abstract

**Purpose:**

To compare patients with and without a history of mental illness on process and outcome measures in relation to prehospital and emergency surgical care for patients with perforated ulcer.

**Methods:**

A nationwide registry-based cohort study of patients undergoing emergency surgery for perforated ulcer. We used data from the Danish Prehospital Database 2016–2017 and the Danish Emergency Surgery Registry 2004–2018 combined with data from other Danish databases. Patients were categorized according to severity of mental health history.

**Results:**

We identified 4.767 patients undergoing emergency surgery for perforated ulcer. Among patients calling the EMS with no history of mental illness, 51% were identified with abdominal pain when calling the EMS compared to 31% and 25% among patients with a history of moderate and major mental illness, respectively. Median time from hospital arrival to surgery was 6.0 h (IQR: 3.6;10.7). Adjusting for age, sex and comorbidity, patients with a history of major mental illness underwent surgery 46 min (95% CI: 4;88) later compared to patients with no history of mental illness. Median number of days-alive-and-out-of-hospital at 90-day follow-up was 67 days (IQR: 0;83). Adjusting for age, sex and comorbidity, patients with a history of major mental illness had 9 days (95% CI: 4;14) less alive and out-of-hospital at 90-day follow-up.

**Conclusion:**

One-third of the population had a history of mental illness or vulnerability. Patients with a history of major mental illness were less likely to be identified with abdominal pain if calling the EMS prior to arrival. They had longer delays from hospital arrival to surgery and higher mortality.

**Supplementary Information:**

The online version contains supplementary material available at 10.1007/s00068-023-02427-1.

## Introduction

People with severe mental illness experience significantly worse surgical outcomes including greater postoperative morbidity, longer stays in hospitals and higher risk of readmissions compared to the general population [1–5]. A few studies have focused on the adverse prognosis for patients with comorbid mental illness undergoing major abdominal emergency surgery [1–4]. An overall underuse of major surgery for patients with severe mental illness has been identified [6], as well of more severe disease presentations upon admission in emergency situations—likely due to patient- and system-related delays in symptom recognition [4].

Peptic ulcer disease is a common condition that both primary care and secondary care providers encounter [7, 8]. The detection of the correlation between the Helicobacter pylori bacteria and peptic ulcer disease has changed the understanding of the disease. Symptoms of peptic ulcer disease are variable and may include abdominal pain, nausea, vomiting and weight loss. Complications of peptic ulcer disease include perforation and bleeding. Recent evidence suggests that hemorrhage is the most common complication [8]. Although the global prevalence of peptic ulcer disease has dramatically decreased in the past decades, the incidence of its complications has remained constant [7]. People with severe mental illness have an increased risk of developing peptic ulcers [9]. Individual factors related to nutrition, socioeconomic status, hygiene and sanitation may explain some of this variation. [7]

Both system- and patient-related factors may contribute to the poorer outcomes in patients with comorbid mental illness. System-related factors may play an important role, since people with mental illness may experience a systematic failure of the healthcare system to prevent, identify and treat physical diseases [10–12]. Stigmatization of patients with mental illness by healthcare professionals could be a contributing factor [13–16]. Patient-related factors may include low socioeconomic status, poor social network, impaired bodily sensation, disturbed perception of pain, poor health literacy, adverse lifestyle factors and physical comorbidities. These factors may all act as mediating factors of mental illness contributing to higher prevalence, later hospital arrival, higher urgency of disease upon admission and poor outcomes. [4, 17–20]

The timing of surgery is crucial in patients with perforated ulcers [21–23]. Investigating initial management, including the prehospital assessment and care, is important to improve quality of care. Also, including patients with different severities of mental illness may improve the understanding of the potential impact of comorbid mental illness. The aim of this study was to compare patients with and without a history of mental illness on process and outcome measures in relation to prehospital and emergency surgical care for patients with perforated ulcer.

## Methods

### Study design and setting

This was a nationwide cohort study combing patient-level data from *Danish Clinical Register of Emergency Surgery* [24] and the *Danish Quality Database for Prehospital Emergency Medical Services* (Prehospital Database) [25] with other nationwide Danish health registries.

The study was reported according to the Strengthening the Reporting of Observational studies in Epidemiology (STROBE) guidelines for observational studies.

A unique ten-digit Civil Personal Register (CPR) number is assigned to all citizens in Denmark. The CPR allows for individual-level record linkage of Danish registers [26]. Both the Register of Emergency Surgery and the Prehospital Database are organized under the publicly funded organization *Danish Clinical Registries* [27]*.* Reporting to the Danish Clinical Registries is mandatory for all hospitals including the emergency medical services (EMS).

The study was conducted in Denmark, which has a population of 5.8 million. The country has five healthcare regions responsible for healthcare services, and each region has an organization responsible for the entire EMS, including the regional Emergency Medical Communication Centre and the ambulance services. The healthcare system is primarily financed through taxation and has free access to healthcare services including access to EMS, general practitioners (GP) and hospital care. Referral from a GP or out-of-hour GP is mandatory prior to all hospital contacts, excluding psychiatric emergencies and patients arriving by ambulance after calling EMS. Emergency surgical care is provided only at public hospitals in Denmark [28]. During the study period, the number of hospitals receiving emergency patients—including emergency surgical patients—was reduced from 44 to 21 hospitals based on recommendations from the Danish Health Authorities in 2007 [29]. Since 2010, hospitals and GPs have access to an online Shared Medication Record [30] though the electronic medical record, providing information on the citizen’s medication including most recent prescriptions and dates of prescription and redemption. Hospitals and GPs also have online access to the hospital-based health records across hospitals providing information on, e.g., diagnosis and discharge letters.

Police or fire brigade personnel answer all EMS calls. Calls identified as health-related are forwarded to one of five public EMCCs [31]. At the EMCC, healthcare professionals handle these calls. The Emergency Medical Communication Centre personnel assess the individual calls using “Danish Index for Emergency Care,” a criteria-based dispatch decision support tool. Each call is assigned a main symptom, which can be selected among 37 standardized mechanisms or symptoms (e.g., symptom card 24 “Abdominal pain or back pain”). Depending on the type and urgency of the symptoms (e.g., sudden severe pain or vomiting red blood), the call is assigned a priority level from “A” to “E.” The highest priority level is urgency level A, corresponding to an immediate response, i.e., dispatching ambulance as category/priority 1.

### Identification of study population

The study population consisted of consecutive patients admitted from January 1, 2004, to December 31, 2018, identified in the Danish Clinical Register of Emergency Surgery. Only patients registered with a diagnosis of perforated ulcer (ICD-10 DK251, − 252, − 255, − 256, − 261, − 262, − 265, − 266, − 271, − 272, − 275, − 276) in combination with a surgical procedural code (any “KJ”-code in the administrative system covering all surgical gastrointestinal interventions) who had the initial surgery performed within 48 h from hospital arrival were included.

**Fig. 1:**
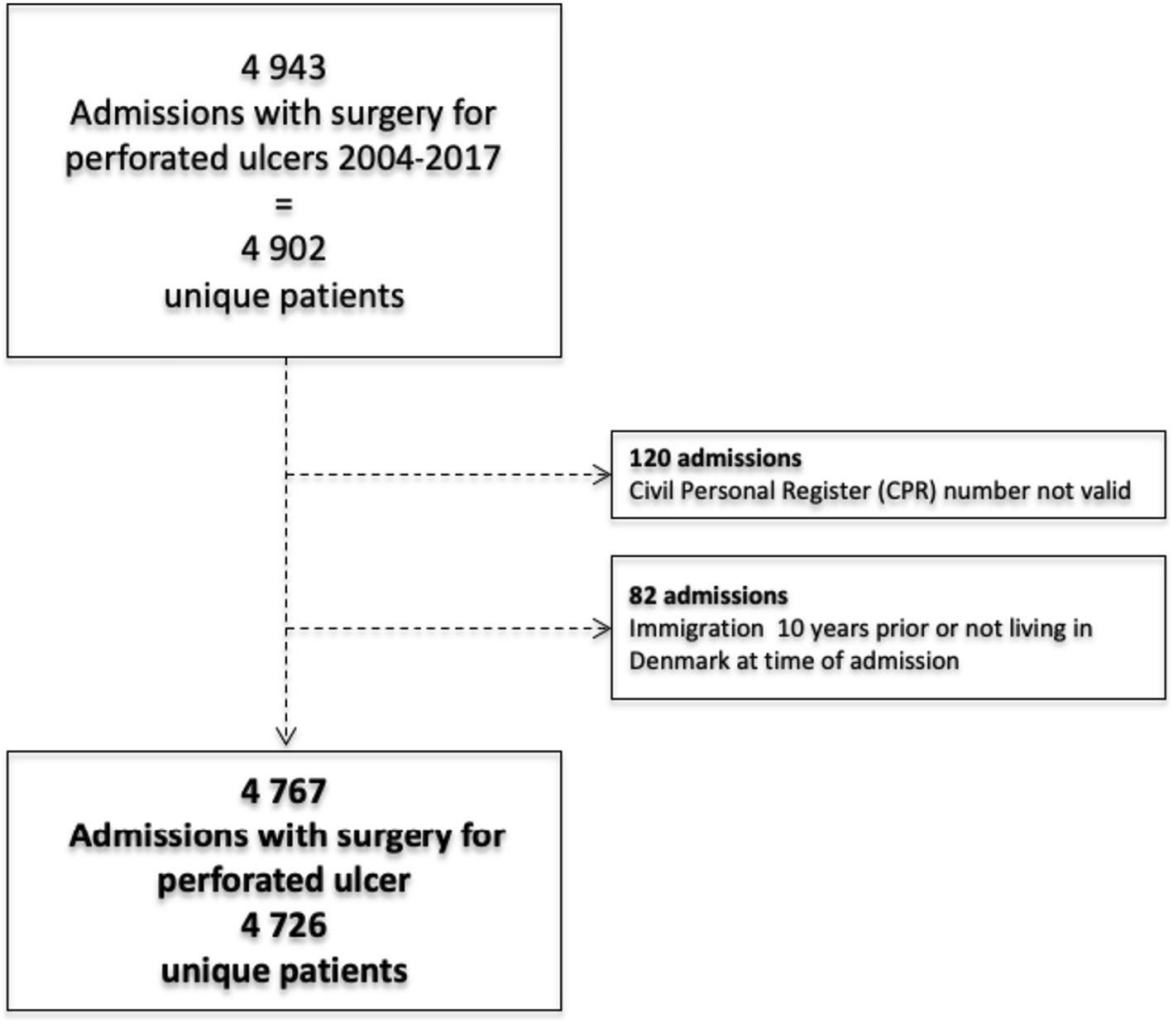
Flowchart of included admissions from Danish Clinical Register of Emergency Surgery 2004–2108 who has surgery performed within 48 h from hospital arrival

Patients were excluded if the patient had immigrated to Denmark less than 10 years prior to the admission or if the patient was not registered as living in Denmark at the time of the admission (Fig. 1). The 10-year cutoff was set due the definition of the exposure, requiring patient’s mental health record for at least 10 years.

The cohort was combined with data from the Prehospital Database covering the years 2016–2017 using the CPR number. If an EMS call was made 72 h prior to hospital arrival, the patient was defined as “EMS patient.” If no EMS call was identified 72 h prior to hospital arrival, the patient was defined as “non-EMS patient.” We chose 72 h, since patients with a history of mental illness are more likely to receive telephone advise or be released at scene, when they call the EMS [32], and may have called more than once or contacted different health professionals prior to admission.

### Ethics

The study was approved by the Danish Data Protection Agency. According to Danish law, approval from an ethical committee is not required for register-based studies.

### Variables

#### Definition of exposure: mental health history

Patients were categorized according to the severity of their mental health history: Major, moderate, minor or none based on information from The Danish National Patient Registry, The Danish National Patient Registry-Psychiatry, The Danish Psychiatric Central Research Register, The National Register of Medicinal Product Statistics and The Danish National Health Service Register. If the patient fulfilled criteria in several categories, the most severe category defined the patient’s exposure.

1. Major mental illness: An inpatient or outpatient contact with schizophrenia or bipolar disorder (ever), or inpatient contacts with severe depression or emotionally unstable personality disorder (within the last 10 years).
2. Moderate mental illness: Other inpatient or outpatient psychiatric contacts with diagnoses of mental illness (other than “major”) or consultations at a private practicing psychiatrist (within the last 5 years).
3. Minor mental illness or vulnerability: None of the above. Reimbursement of at least two prescription of selected drugs (antidepressants or benzodiazepines) or two or more sessions of talk therapy or psychometric testing in a primary care setting or referral to a private psychologist (within the past 12 months).
4. No mental illness: None of the above.

While there is no gold standard for categorizing mental illnesses, it is generally accepted that major mental illness includes schizophrenia and bipolar disease [10]. Hospital-based diagnoses are often used to identify mental illness in registry-based studies [33] and is considered to be more severe than mental health conditions requiring community-based services only (e.g., GP or psychologist services). Please see Supplementary Table 1, for details on included ICD-10 diagnoses, other codes (e.g., ATC codes) and data sources. Please see Supplementary Table 2 for information about timing of most recent mental health disease-related activity.

#### Definition of outcomes: Prehospital care and emergency surgical care

We defined prehospital care and emergency surgical care using process performance measures and outcome measures from the Danish Prehospital Database and the Danish Clinical Register of Emergency Surgery. See Table 1 for details on each measure. The Danish guidelines for emergency surgical care are in accordance with the guidelines from World Society of Emergency Surgery (WSES). [8]

**Table 1:** Performance measures from the Prehospital Database and the Danish Clinical Register of Emergency Surgery

No	Performance measures	Period available
Prehospital process measures		
1 EMS response	Proportion of patients assigned the highest level of urgency (urgency level A), when calling the EMS	2016–2017
2 Symptoms	Proportion of patients recognized with abdominal pain, when calling the EMS	2016–2017
Emergency surgical process measures		
3 Antibiotics	Administration of i.v. antibiotics within 1 h from hospital arrival	2014–2018
4 Preoperative risk stratification	Preoperative risk assessment using the PULP (Peptic Ulcer Perforation) score^33^	2014–2018
5 Preoperative optimization	Preoperative treatment with oxygen and i.v. fluids/blood	2014–2018
6 Surgery delay	Time from hospital arrival to surgery	2004–2018
Outcome measures		
7 Mortality	Death within 30 days of primary surgical procedure	2004–2018
8 Combined outcome	Days alive-and-out-of-hospital at 90 days	2004–2018

While we used the measures from the Emergency Surgery Registry 2004–2018, some measures have been updated since 2018 to reflect new evidence and best clinical practice within the field. Since 2019, the registry includes other high risk surgical patients than patients with perforated ulcers. Also, measures have been added regarding, e.g., timing of CT abdomen, measurement of serum lactate and timing of preoperative optimization. These measures were not available when we received our dataset in 2019. However, the original measures from 2004 to 2018 still reflect the provision of the core processes of early surgical care and emergency care for patients with perforated ulcers.

For the combined outcome “Days alive and out-of-hospital,” we applied the same methodology as described by colleagues [34]: At 90-day follow-up post-surgery, we subtracted the length of stay of the initial admission and all readmissions within this time period to calculate the number of days alive and out of hospital. If a patient died within the 90 days, number of days alive and out of hospital was defined as 0 days.

### Covariates

Covariates available for all patients included: Age, sex, comorbidity (Charlson comorbidity index), American Society of Anaesthesiologists Classification (ASA) score, type of surgery, smoking status, social position, cohabitation status and drug abuse. Detailed definitions of covariates and data sources are presented in Supplementary Table 3.

Supplementary Fig. 1 provides a directed acyclic graph (DAG) presenting covariates as confounding factors or mediators.

### Statistical methods

We compared all measures according to the severity of mental illness using the group with no history of mental illness as a reference.

For the time delays (time to surgery and time to antibiotics), we compared the exposure groups applying robust linear regression based on Huber and biweight iterations, as implemented in the Stata routine *rreg*. The purpose of this regression was to limit the effect of serious outliers. Please see boxplot Supplementary Fig. 2 for the distributions of time to surgery and time to antibiotics including outliers. We performed an additional analysis comparing medians and 75 percentile to further explore the differences between groups. Moreover, we estimated a concordance index (c-index), which is rank-based statistics ranging from zero to one. Thus, the c-index is a measure of how well the exposure groups discriminate the outcome, with c-index equal 0.5 means zero discrimination, while a c-index equal zero or one means full discrimination. Adjusted c-indices were estimated using inverse-probability-of-treatment-weights, i.e., first, the probability of being exposed delayed surgery is calculated, given an individual’s characteristics. Second, weights are calculated (the inverse of the propensity score). The application of weights creates a pseudopopulation in which confounders are equally distributed across exposed and unexposed groups.

For the analysis of differences in days-alive-and-out-of-hospital, we used linear regression. For the binary outcomes, we calculated a Risk Ratio (RR) using Poisson regression. To relax the assumption of independence for recurring subjects, we applied the clustered Huber variance estimator to these regressions.

All estimates were calculated as unadjusted and adjusted for age and sex (model 1) and for age, sex and Charlson comorbidity index (model 2). We also performed additional analyses adjusting for age, sex, Charlson comorbidity index and ASA score (model 3). Please see Supplementary Fig. 1: Directed acyclic graph for consideration of covariates as confounders or mediators. Also, since both mortality and the proportion of patients with a history of mental illness decreases in the study period (Supplementary Table 5), analyses regarding days-alive-and-out-of-hospital and 30-day mortality were adjusted for calendar year.

We implemented restricted cubic splines for age with three knots at quantile 0.1, 0.5 and 0.9.

Missing ASA scores and time to surgery were imputed implementing multiple imputation chained equations using 20 imputation sets. All outcomes, exposures and covariates were included as predictors. For the c-index, we used singular value imputation by only using the first imputation set of the multiple value imputation.

Statistical analyses were performed using Stata 16 (StataCorp. 2019. Stata Statistical Software: Release 16. College Station, TX: StataCorp LLC.).

## Results

We identified 4.767 admissions with patients undergoing emergency surgery for perforated ulcer. Of these 25% had a history of minor mental illness or vulnerability, while 4% and 4% had a history of moderate or major mental illness, respectively. Characteristics of the population are shown in Table 2. A total of 84 patients (2%) were missing timestamp (hours and minutes) (supplementary Table 4). Please see Table 2 regarding missing values for each covariate.

**Table 2:** Characteristics of the patients undergoing surgery for perforated ulcers within 48 h from hospital arrival 2004–2018 in Denmark according to severity of mental health history

	No history of mental illness *N* = 3180	History of minor mental illness *N* = 1197	History of moderate mental illness *N* = 199	History of major mental illness or vulnerability *N* = 191	Missing
Sex, male	52%	31%	44%	45%	0
Age, median (IQR)	70 (58;81)	76 (65;84)	60 (50;73)	67 (56;76)	0
Charlson comorbidity (cci) 0 1–2 3–4 5 +	116 (4%)	26 (2%)	10 (5%)	7 (4%)	0
	2087 (66%)	686 (57%)	125 (63%)	122 (64%)	
	637 (20%)	303 (25%)	44 (22%)	34 (18%)	
	340 (11%)	182 (15%)	20 (10%)	28 (15%)	
ASA score 1 2 3 4–5	654 (21%)	89 (8%)	38 (19%)	23 (12%)	73
	1114 (36%)	398 (34%)	73 (37%)	65 (35%)	
	1026 (33%)	494 (42%)	69 (35%)	71 (38%)	
	343 (10%)	191 (16%)	17 (9%)	32 (15%)	
Substance abuse, alcohol/other drugs	243 (8%)	148 (12%)	69 (35%)	62 (32%)	0
Smoking Current Previous Never	1596 (56%)	556 (53%)	121 (68%)	115 (65%)	492
	447 (16%)	174 (17%)	20 (11%)	26 (15%)	
	828 (29%)	318 (30%)	38 (21%)	36 (20%)	
Cohabitation, Living alone	1,674 (53%)	756 (63%)	126 (63%)	142 (74%)	50
Level of education, individual level High Medium Low	363 (13%)	122 (11%)	31 (16%)	29 (16%)	477
	1059 (37%)	344 (32%)	68 (36%)	65 (36%)	
	1435 (50%)	597 (56%)	89 (47%)	88 (48%)	
Household income Above country average Below country average Poverty (national definition*)*	909 (30%)	306 (27%)	63 (32%)	37 (20%)	781
	2021 (66%)	819 (72%)	115 (59%)	139 (76%)	
	130 (4%)	19 (2%)	17 (9%)	8 (4%)	
Household adherence to workforce (best in house) Working/education Retirement (age-related) Public benefits	803 (26%)	159 (13%)	40 (20%)	25 (13%)	92
	1897 (61%)	874 (73%)	79 (40%)	101 (53%)	
	396 (13%)	160 (13%)	77 (39%)	64 (34%)	
Type of surgery* Laparotomy Laparoscopy Laparoscopy converted to laparotomy	409 (29%)	143 (36%)	28 (34%)	28 (36%)	11*
	614 (44%)	156 (39%)	40 (49%)	37 (47%)	
	374 (27%)	103 (26%)	14 (17%)	13 (17%)	
Time from onset of symptoms to hospital arrival** median, IQR	307 min (154;716)	290 min (123;569)	356 min (171;1492)	658 min (269;4009)	448**

^*^available only 2012–2018. Number of missing are the number within this period

^**^available only 2014–2018. Number of missing are the number within this period

*ASA* American Society of Anaesthesiologists Classification, *IQR* inter-quartile range

Considering patients admitted 2016–2017 (*n* = 640), 153 (24%) called the EMS prior to admission. While 48% of patients with no history of mental illness were assigned an immediate response (Level A) when calling the EMS, only 38% and 25% of patient with a history of moderate or major history of mental illness, respectively, were assigned this level of urgency (Table 3). Among patients with no history of mental illness, 51% were identified with abdominal or back pain when calling the EMS (Table 3). This number was 40%, 31% and 25% for patients with a history of minor, moderate and major mental illness, respectively (Table 3).

**Table 3:** Contact to emergency medical services (EMS), presenting symptoms and proportion receiving a response with the highest level of urgency (level A) for patients undergoing surgery for perforated ulcers 2016–2017 according to mental health history

	No history of mental illness (*n* = 360)	History of minor mental illness (*n* = 89)	History of moderate mental illness (*n* = 18)	History of major mental illness or vulnerability (*n* = 17)
Proportion calling the EMS 72 h prior to hospital arrival, Proportion	33%	27%	44%	24%
Proportion receiving an EMS response with the highest level of urgency, Proportion	48%	67%	38%	25%
Presenting symptom, proportions				
Abdominal or back pain	51%	40%	31%	25%
Other (non-abdominal) symptom	34%	48%	38%	62%
Unclear problem	15%	12%	31%	13%

**Fig. 2:**
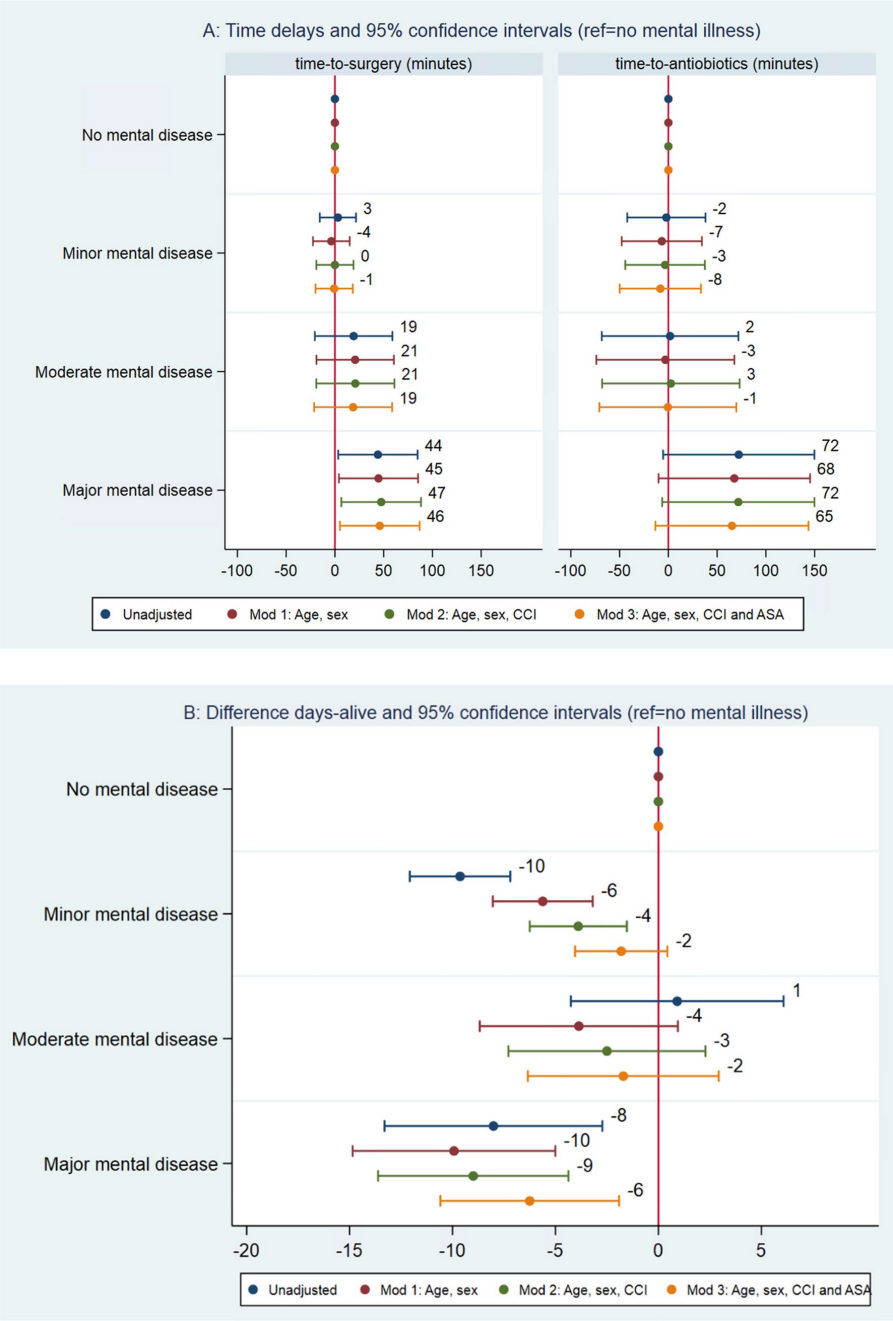
Time differences (minutes) regarding time to surgery (2**A** left) and time to antibiotics (2**A** right) and differences in days-alive-and-out-of hospital (2**B**) between patients with a history of mental illness. Patient with no history of mental illness as reference. *CCI* Charlson comorbidity index, *ASA* American Society of Anaesthesiologists Classification

For all patients in the cohort (2004–2018), the median time from hospital arrival to surgery was 6.0 h (IQR: 3.6;10.7) (Supplementary Table 4). Adjusting for age, sex and comorbidity, patients with a history of major mental illness underwent surgery 46 min (95% CI: 4;88) later compared to patients with no history of mental illness (Fig. 2a). Differences in medians and 75 percentile and c-indices can be found in Supplementary Table 6. The c-indices revealed a statistically significant, but small discrimination in treatment delay for patients with a history of major illness compared to no history of mental illness. The regression analyses on 75% quartile showed that for a portion of patients with a history of moderate or major mental illness, surgery was delayed compared to the patients with no history of mental illness.

Considering patients admitted 2014–2018, the median time from hospital arrival to antibiotics was 207 min (IQR: 90;423). (Supplementary Table 4). Adjusting for age, sex and comorbidity, patients with a history of major mental illness received antibiotics 72 min (95% CI: − 10;148) later, compared to patients with no history of mental illness (Fig. 2a). While differences were not statistically significant, notable differences were observed for patients with moderate and major mental health history, with a large proportion of patients receiving antibiotics very late (e.g., 75 percentile above 500 min) (Box plot supplementary Fig. 2 and Supplementary Table 4). Differences in medians, 75 percentile and c-indices can be found in Supplementary Table 6.

We found no differences regarding preoperative risk assessment nor preoperative hemodynamical optimization between groups (Supplementary Table 4)30-day mortality for all patients was 23% and number of days-alive-and-out-of-hospital at Days 90 was 67 days (IQR: 0;83) (Supplementary Table 4). Adjusting for age, sex and comorbidity, patients with a history of major mental illness had a higher risk of death at 30-day risk ratio 1.43 (95% CI: 1.14;1.80) and 9 days (95% CI: 4;14) less alive and out-of-hospital at 90-day follow-up, compared to patients with no history of mental illness (Fig. 2b). Patients with a minor history of mental illness had a higher risk of death at 30-day Risk Ratio 1.16 (95% CI: 1.04;1.29) and 4 days (IQR: 2;6) less alive and out-of-hospital at 90-day follow-up (Fig. 2b). During the study period, 30-day mortality decreased and the median number of days-alive-and-out-of-hospital at 90-day follow-up increased (Supplementary Table 5). We found no significant differences in 30-day mortality of days-alive-and-out-of-hospital among patients with a history of moderate mental illness (Fig. 2b).

## Discussion

In this nationwide study, we found that patients with a history of moderate or major mental illness were less likely to be assigned an immediate response and less likely to be recognized with abdominal pain, if calling the EMS prior to hospital arrival. Further, we found that patients with a history of major mental illness underwent surgery and had antibiotics later, compared to patients with no history of mental illness. Patients with a history of major mental illness or minor mental illness/vulnerability also had a higher risk of 30-day mortality and had significantly fewer days alive-and-out-of-hospital at 90-day after surgery.

Our finding of a median of six hours delay from hospital arrival to surgery—regardless of mental health history—is long. This delay is longer than the national recommendations, and both local and national initiatives have been developed to decrease surgical delays by introducing, i.e., multi-disciplinary protocols [35–37]. The even longer delays for patients history of major mental illness is concerning and may be partly explained by the fact, that these patients were more likely to have atypical or unclear presentation when calling the EMS. This is likely to delay both antibiotics and surgery. Atypical and unclear presentation may be due to an altered pain response observed in patient with major mental illness [2, 4, 20, 38]. This may cause the longer patient delay that we found from onset of symptoms to hospital arrival—especially in the major group—if the patient is not aware of the urgency of the symptoms.

The importance of a bystander has been described in other time-dependent diseases (i.e., stroke [39]) and may be true for abdominal emergencies as well. However, an important system-related factor may also be the role of stigma and diagnostic overshadowing (e.g., *assuming that a symptom is due to coexisting mental health condition rather than exploring the cause of the patient's symptoms)* [13–16, 20]*.* Another Danish study, investigating EMS responses among selected emergency diseases, found an overall tendency among patients with perforated ulcers, to receive a lower urgency of EMS response compared to other time-dependent diseases, i.e., only 64% of patients received an immediate EMS response [40]. Hence, recognizing the severity of symptoms in perforated peptic ulcers may be difficult for both patients and health professionals, regardless of mental health history. Also, considering the low proportion of patients even calling the EMS prior to admission, compared to other time-dependent diseases, i.e., stroke and myocardial infarction [41] identifying these vulnerable subgroups may be a particular challenge, especially as patients with severe mental illness also have been reported to have an increased risk of developing peptic ulcers [9]. Ensuring correct system response for these high-risk patients is crucial. Staff competencies, stigma within the healthcare system and organizational health literacy including decision support tools supporting the health professionals dealing with vulnerable and high-risk patients must be addressed.

Our finding of worse outcome among patients with a history of moderate or major mental illness are in accordance with studies investigating different types of urgent surgery [1–5]. These studies also find greater postoperative morbidity, longer stays in hospitals and higher risk of readmissions [1–5]. Our finding of a higher 30-day mortality among patients with a history of major mental illness is in accordance with a study investigating outcomes in patients undergoing emergency laparotomy (for other causes than perforated ulcers) according to socioeconomic status. They found poorer outcome and higher 30-day mortality for patients with the most disadvantaged socioeconomic position patients compared to patients with a more advantaged socioeconomic position [17]. In our study, patients with a history of major mental illness also had the most disadvantaged socioeconomic position. The complex interplay between patient-related factors [4, 17–20] and system-related factors [10–16] within the healthcare system is likely to mediate or exacerbate poor outcome within this subgroup. Patient-related factors such as social network, individual health literacy, adverse lifestyle factors including nutrition and hygiene may also contribute to surgery-related complications and ability to participate in follow-up.

Our finding of higher ASA scores among patients with a history of either minor or major mental illness may also explain some of this variation. The higher ASA scores may be due to both higher burden of comorbidity, substance abuse, functional status, level of independency, as well as acute derangement. In addition, the poorer outcome among patients with a history of minor mental illness is likely due to higher age burden of comorbidity, which may also explain why we do not find a dose–response relationship between the different severities of mental illness and our outcomes. That is, patients with a history of minor mental illness experienced the worst outcomes, while patients with a history of moderate mental illness experienced almost equal outcomes, compared to patients with no history of mental illness.

In a larger perspective, our finding of fewer days-alive-and-out-of-hospital is in accordance with existing studies describing lost life years and excess mortality in general among patients with mental illness [10, 42–45]. While other studies investigated lost life years over a lifetime combing the contributions from emergency and chronic diseases, our study contributes with knowledge regarding lost life years in relation to urgent care.

### Strengths and limitations

The data sources used in this paper represent a key strength with an overall high completeness and accuracy of the Danish databases and registries. However, several limitations should be considered.

First, although the study was based on population-based registries and the Danish Emergency Surgery Registry has been shown to covers 89% of all admissions with perforated ulcers [46], patients with mental illness may be more likely to have undetected events of perforated ulcers [6, 20, 38]. Hence, our results can only be generalized to patients, who are admitted to the hospital, recognized and treated for a perforated ulcer.

Also, information bias may have been introduced. The definition of exposure is a proxy measure of mental illness. It is defined based on consensus between local experts since there is no gold standard for these definitions. In particular, the definition of the minor illness group may be prone to misclassification, since these measures may express a state of mental vulnerability rather than mental illness. Supplementary Table 2 provides information on available measures of recent mental health disease-related activity.

Our estimates may also be skewed due to confounding. While we adjusted for some factors (e.g., age and sex), other factors (CCI and ASA score) are most likely mediators. Adjusting our estimates for both CCI and ASA score may remove some of the effect of having a history of mental illness. Either ways, residual confounding is expected to persist in these very heterogenic groups. In particular, patients with a minor history of mental illness may have an inherent frailty (considering their higher age, burden of comorbidity combined with mental illness) that we were not able to adjust for using the available measures.

Also, due to the small number of patients, especially in the moderate and major groups, estimates regarding antibiotics based on data from 2014 to 2018 had a moderate to low precision.

## Conclusion

One-third of patients admitted with a perforated ulcer had a history of mental illness or vulnerability. Patients with a history of major mental illness had longer delays from symptom onset to hospital arrival. Patients with a history of major or moderate mental illness were less likely to be recognized with abdominal pain and less likely to be assigned the highest levels of urgency, if calling the EMS prior to arrival. Patients with a history of major mental illness had longer delays from symptom and from hospital arrival to surgery and showed a tendency to receive antibiotics later, compared to patients with no history of mental illness. They also had a higher risk of 30-day mortality and has less days alive-and-out-of-hospital at 90-day follow-up.

### Supplementary Information

Below is the link to the electronic supplementary material.

Supplementary file1 (DOCX 490 KB)

## Data Availability

Data cannot be shared publicly because of Danish legislation. Data can be accessed through the Danish Health Data Authority and Statistics Denmark for researchers at authorized institutions. Information on data access is available online (http://sundhedsdatastyrelsen.dk/da/forskerservice). Access to data requires approval from the Danish Data Protection Agency (https://www.datatilsynet.dk/english/legislation. The authors did not have special access privileges to these data.

## Abbreviations

ASA: American Society of Anaesthesiologists Classification
EMS: Emergency medical services
GP: General practitioner

## References

[1] McBride KE, Solomon MJ, Bannon PG, Glozier N, Steffens D (2021). Surgical outcomes for people with serious mental illness are poorer than for other patients: a systematic review and meta-analysis. Med J Aust.

[2] Tsay JH, Lee CH, Hsu YJ, Wang PJ, Bai YM, Chou YJ (2007). Disparities in appendicitis rupture rate among mentally ill patients. BMC Public Health.

[3] Liao CC, Shen WW, Chang CC, Chang H, Chen TL (2013). Surgical adverse outcomes in patients with schizophrenia: a population-based study. Ann Surg.

[4] Nishihira Y, McGill RL, Kinjo M (2017). Perforated appendicitis in patients with schizophrenia: a retrospective cohort study. BMJ Open.

[5] Copeland LA, Zeber JE, Pugh MJ, Mortensen EM, Restrepo MI, Lawrence VA (2008). Postoperative complications in the seriously mentally ill: a systematic review of the literature. Ann Surg.

[6] Copeland LA, Zeber JE, Sako EY, Mortensen EM, Pugh MJ, Wang CP (2015). Serious mental illnesses associated with receipt of surgery in retrospective analysis of patients in the veterans health administration. BMC Surg.

[7] Abbasi-Kangevari M, Ahmadi N, Fattahi N, Rezaei N, Malekpour MR, Ghamari SH (2022). Quality of care of peptic ulcer disease worldwide: a systematic analysis for the global burden of disease study 1990–2019. PLoS ONE.

[8] Tarasconi A, Coccolini F, Biffl WL, Tomasoni M, Ansaloni L, Picetti E (2020). Perforated and bleeding peptic ulcer: Wses guidelines. World J Emerg Surg.

[9] Momen NC, Plana-Ripoll O, Agerbo E, Benros ME, Borglum AD, Christensen MK (2020). Association between mental disorders and subsequent medical conditions. N Engl J Med.

[10] Liu NH, Daumit GL, Dua T, Aquila R, Charlson F, Cuijpers P (2017). Excess mortality in persons with severe mental disorders: a multilevel intervention framework and priorities for clinical practice, policy and research agendas. World Psychiatry.

[11] OECD/EU (2016) Health at a glance: Europe 2016 – state of health in the eu cycle

[12] De Hert M, Correll CU, Bobes J, Cetkovich-Bakmas M, Dan C, Asai I (2011). Physical illness in patients with severe mental disorders. I. prevalence, impact of medications and disparities in health care. World Psychiatry.

[13] McBride KE, Solomon MJ, Steffens D, Bannon PG, Glozier N (2019). Mental illness and surgery: do we care?. ANZ J Surg.

[14] McBride KE, Solomon MJ, Lambert T, O'Shannassy S, Yates C, Isbester J (2021). Surgical experience for patients with serious mental illness: a qualitative study. BMC Psychiatry.

[15] Solvhoj IN, Kusier AO, Pedersen PV, Nielsen MBD (2021). Somatic health care professionals' stigmatization of patients with mental disorder: a scoping review. BMC Psychiatry.

[16] van Nieuwenhuizen A, Henderson C, Kassam A, Graham T, Murray J, Howard LM (2013). Emergency department staff views and experiences on diagnostic overshadowing related to people with mental illness. Epidemiol Psychiatr Sci.

[17] Poulton TE, Moonesinghe R, Raine R, Martin P (2020). National emergency laparotomy audit project t. socioeconomic deprivation and mortality after emergency laparotomy: an observational epidemiological study. Br J Anaesth.

[18] Eugene N, Oliver CM, Bassett MG, Poulton TE, Kuryba A, Johnston C (2018). Development and internal validation of a novel risk adjustment model for adult patients undergoing emergency laparotomy surgery: The national emergency laparotomy audit risk model. Br J Anaesth.

[19] Hasselager RB, Lohse N, Duch P, Moller MH (2016). Risk factors for reintervention after surgery for perforated gastroduodenal ulcer. Br J Surg.

[20] Kallur A, Yoo E, Bien-Aime F, Ammar H (2022). Diagnostic overshadowing and pain insensitivity in a schizophrenic patient with perforated duodenal ulcer. Cureus.

[21] Buck DL, Vester-Andersen M, Moller MH (2013). Danish clinical register of emergency S. surgical delay is a critical determinant of survival in perforated peptic ulcer. Br J Surg.

[22] Moller MH, Adamsen S, Thomsen RW, Moller AM (2011). Peptic ulcer perforation trial g Multicentre trial of a perioperative protocol to reduce mortality in patients with peptic ulcer perforation. Br J Surg.

[23] Moller MH, Norgard BM, Mehnert F, Bendix J, Nielsen AS, Nakano A (2009). preoperative delay in patients with peptic ulcer perforation: a clinical audit from the danish national indicator project. Ugeskr Laeger.

[24] Moller MH, Larsson HJ, Rosenstock S, Jorgensen H, Johnsen SP, Madsen AH (2013). Quality-of-care initiative in patients treated surgically for perforated peptic ulcer. Br J Surg.

[25] Christensen EF, Berlac PA, Nielsen H, Christiansen CF (2016). The danish quality database for prehospital emergency medical services. Clin Epidemiol.

[26] Schmidt M, Schmidt SAJ, Adelborg K, Sundboll J, Laugesen K, Ehrenstein V (2019). The danish health care system and epidemiological research: from health care contacts to database records. Clin Epidemiol.

[27] Sorensen HT, Pedersen L, Jorgensen J, Ehrenstein V (2016). Danish clinical quality databases - an important and untapped resource for clinical research. Clin Epidemiol.

[28] Liljendahl MS, Gogenur I, Thygesen LC (2020). Emergency laparotomy in denmark: a nationwide descriptive study. World J Surg.

[29] Sundhedsstyrelsen. Styrket akutberedskab - et nyt planlægningsgrundlag for det regionale akutberedskab [danish health authorities. Improving emergency care - planning emerency care on a regional level]. 2007

[30] Danish health data authority. “Digital health solutions - shared medication record”. https://sundhedsdatastyrelsen.Dk/da/english/digital_health_solutions (last accessed july 8th 2022)

[31] Mikkelsen S, Lassen AT (2020). The danish prehospital system. Eur J Emerg Med.

[32] Mackenhauer J, Valentin JB, Mikkelsen S, Steinmetz J, Vaeggemose U, Christensen HC (2021). Emergency medical services response levels and subsequent emergency contacts among patients with a history of mental illness in denmark: a nationwide study. Eur J Emerg Med.

[33] Plana-Ripoll O, Pedersen CB, Agerbo E, Holtz Y, Erlangsen A, Canudas-Romo V (2019). A comprehensive analysis of mortality-related health metrics associated with mental disorders: a nationwide, register-based cohort study. Lancet.

[34] Spurling LJ, Moonesinghe SR, Oliver CM (2022). Validation of the days alive and out of hospital outcome measure after emergency laparotomy: a retrospective cohort study. Br J Anaesth.

[35] Cihoric M, Kehlet H, Hojlund J, Lauritsen ML, Kanstrup K, Foss NB (2023). Perioperative changes in fluid distribution and haemodynamics in acute high-risk abdominal surgery. Crit Care.

[36] Timan TJ, Sernert N, Karlsson O, Prytz M (2020). Smash standardised perioperative management of patients operated with acute abdominal surgery in a high-risk setting. BMC Res Notes.

[37] Tengberg LT, Bay-Nielsen M, Bisgaard T, Cihoric M, Lauritsen ML, Foss NB (2017). Multidisciplinary perioperative protocol in patients undergoing acute high-risk abdominal surgery. Br J Surg.

[38] Retamero C, Paglia C (2012). When patients do not hurt: Silent acute abdomen in a patient with schizophrenia. Gen Hosp Psychiatry.

[39] Iversen AB, Blauenfeldt RA, Johnsen SP, Sandal BF, Christensen B, Andersen G (2020). Understanding the seriousness of a stroke is essential for appropriate help-seeking and early arrival at a stroke centre: a cross-sectional study of stroke patients and their bystanders. Eur Stroke J.

[40] Bonnesen K, Friesgaard KD, Boetker MT, Nikolajsen L (2018). Prehospital triage of patients diagnosed with perforated peptic ulcer or peptic ulcer bleeding: an observational study of patients calling 1-1-2. Scand J Trauma Resusc Emerg Med.

[41] Sovso MB, Christensen MB, Bech BH, Christensen HC, Christensen EF, Huibers L (2019). Contacting out-of-hours primary care or emergency medical services for time-critical conditions - impact on patient outcomes. BMC Health Serv Res.

[42] Weye N, Momen NC, Christensen MK, Iburg KM, Dalsgaard S, Laursen TM (2020). Association of specific mental disorders with premature mortality in the danish population using alternative measurement methods. JAMA Netw Open.

[43] Plana-Ripoll O, Weye N, Momen NC, Christensen MK, Iburg KM, Laursen TM (2020). Changes over time in the differential mortality gap in individuals with mental disorders. JAMA Psychiat.

[44] Nordentoft M, Wahlbeck K, Hallgren J, Westman J, Osby U, Alinaghizadeh H (2013). Excess mortality, causes of death and life expectancy in 270,770 patients with recent onset of mental disorders in denmark, finland and sweden. PLoS ONE.

[45] Collaborators GBDMD (2022). Global, regional, and national burden of 12 mental disorders in 204 countries and territories, 1990–2019: a systematic analysis for the global burden of disease study 2019. Lancet Psychiatry.

[46] The Danish Clinical Register of Emergency Surgery. 2019. https://www.rkkp.dk/kvalitetsdatabaser/databaser/akut-kirurgi-databasen/

