# Supplementary material for: Disparities in prehospital and emergency surgical care among patients with perforated ulcers and a history of mental illness: a nationwide cohort study
Source: Eur J Trauma Emerg Surg. 2024 Feb 14;50(3):975–85. doi: 10.1007/s00068-023-02427-1 (PMC11249459; doi:10.1007/s00068-023-02427-1)
Supplement: Supplementary file 1 — Supplementary file1 (DOCX 490 KB) [file 68_2023_2427_MOESM1_ESM.docx]

# SUPPLEMENTARY


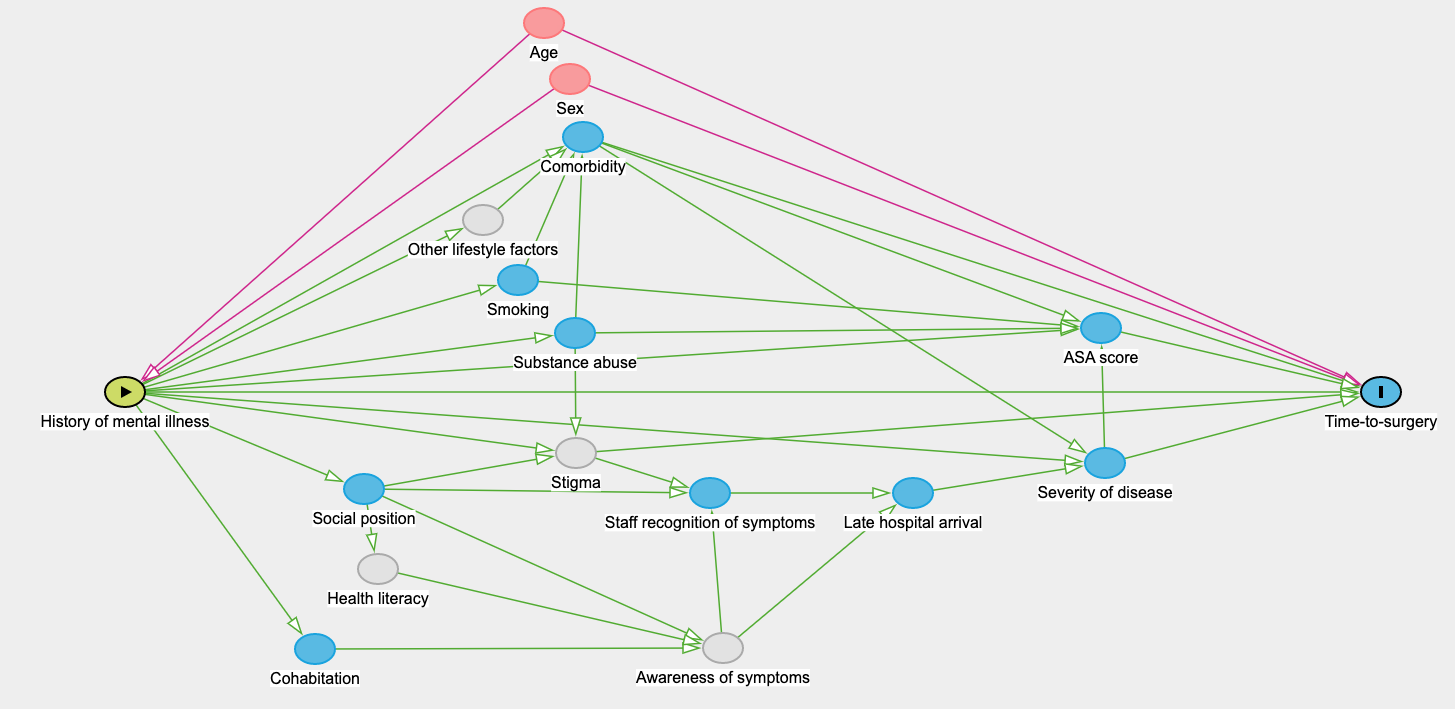
**Supplementary Figure 1.** Directed acyclic graph (DAG) classifying co-variates as confounders (e.g., sex and age) or mediators (e.g., co-morbidity, ASA-score, cohabitation, social position and substance abuse) or unmeasured/unobserved (health literacy, stigma and other lifestyle factors). Example with the association between history of mental illness and time-to-surgery.

### Supplementary Figure 2: Box plots illustrating outliers in the analysis regarding time-to-antibiotics and time-to-surgery


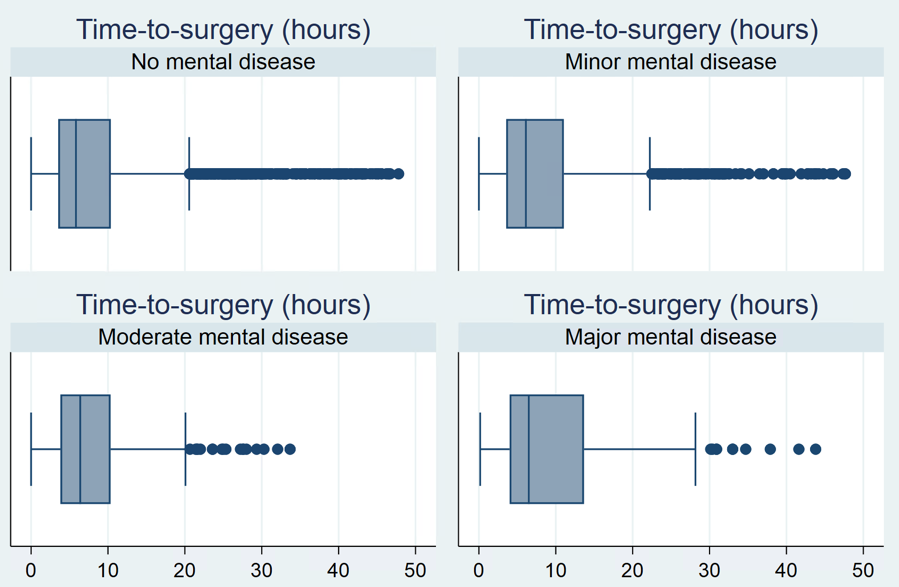


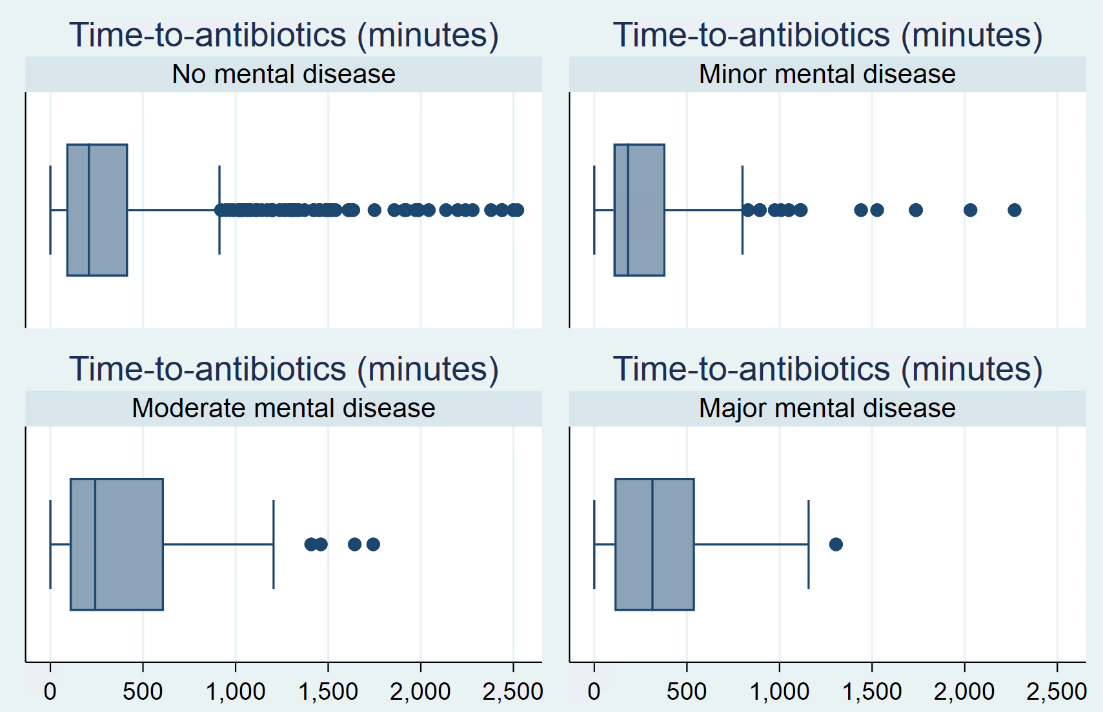


### Supplementary Table 1: Consensus definition of mental illness and – vulnerability

These groups were defined based on consensus among local experts in clinical epidemiology, psychiatry, social medicine and family medicine/general practice, supported by knowledge of patient groups experiencing the highest excess mortality.^30, 41^

|  | **Definition** | **Data Source** |
| --- | --- | --- |
| **History of major mental illness** | *Any in- or outpatient contact (ever)*  **ICD-10** DF20-22 and/or **ICD-8** 295: Schizophrenia  **ICD-10** DF30-31 and/or **ICD-8** 296: Bipolar disease  *Within 10 years prior to admission: In-patient contacts > 2 days*  **ICD-10** DF32-34: Uni-polar depression  **ICD-10** DF60.4: Emotional personality disorder | *The Danish National Patient Registry*^44^  *The Danish National Patient Registry-Psychiatry*  *The Danish Psychiatric Central Research Register*^45^  All three registries contain patient-level data on diagnoses according to the ICD-coding system for all hospital contacts including emergency department visits. |
| **History of moderate mental illness** | *Within 5 years prior to admission: In- or outpatient contact*  **ICD-10** DF23-29: Psychotic disorders, DF38-39: Other affective disorders, DF40-48: Anxiety, and other nonpsychotic disorders, F50-F59 Behavioral syndromes, F60-F69 Disorders of adult personality, F70-F79 Intellectual disabilities, F80-F89 Pervasive and developmental disorders, F90-F98 Behavioral and emotional disorders with onset usually occurring in childhood and adolescence, or F99 Unspecified mental disorder  Regarding ICD-10 DF32-34 and DF60.4: only *out-patient contacts and in-patient stays < 2 days* |  |
|  | **OR** *Within 5 years prior to admission*  **One or more consultations with a private psychiatrist** C_SPECIALE =24, 26 or 35 |  |
| **History of minor mental illness/ vulnerability** | *Within 1 year prior to admission*  **Two or more consultations with a psychologist**  C_SPECIALE =63 |  |
|  | **OR Two or more sessions with talk therapy** OR **Two or more psychometric tests at general practitioner**  C_SPECIALE = 80 *and* C_YDELSESNR =2149 or 6101 or 4003 or 4021 or 4050 or 4063 or 4106 or 4247 or 4248 or 4249 |  |
|  | **OR Two or more redeemed prescriptions**  Benzodiazepiner: MN03AE, -05BA, -05CD or -05CF  Antidepressants: MN06A02 or -06AX26 | *The* *National Register of Medicinal Product Statistics*^46^ contains data on individual-level redeemed prescriptions according to Anatomical Therapeutic Chemical (ATC). |
| **No history of mental illness** | None of the above | |

### Supplementary Table 2: Available measures of most recent* disease activity prior to admission with perforated ulcer

|  | **Most recent* contact in a *psychiatric* hospital**  *(any ICD10 DF-diagnosis)* | **Most recent* private psychiatrist** | **Most recent* redeemed prescription benzo-diazepines/ antidepressant** | **Most recent* session talk therapy, psychometrics or psychologist** |
| --- | --- | --- | --- | --- |
| **Major** | 509 days [108;1510] | 1488 days [700;2606] | 28 days  [12;170] | 699 days [273;1457] |
| **Moderate** | 555 days [159;1113] | 570 days [75;1291] | 35 days  [11;141] | 564 days [148;1083] |
| **Minor** | Not relevant | Not relevant | 28 days  [10;63] | 118 days  [44;230] |

** Disease activity within 10 years for major, 5 years for moderate, 1 year for minor*

**Supplementary Table 3: Co-variates: Definitions and data sources**

| **Variable** | **Definition** | **Data source** | |
| --- | --- | --- | --- |
| **Sex** | Defined as male or female according to the last digit in the civil registration number. Even numbers are used for women, odd numbers for men. If an individual changes gender (psychically and legally), they will be provided with a new number from the Danish Civil Registration System. | *The Danish Civil Registration System*^47^ is an administrative register established in 1968. It contains individual-level information on all persons residing in Denmark. It is updated daily with information on migration and vital status. The data in the Civil Registration System is virtually complete and have high accuracy, which allows for nationwide cohort studies with virtually complete long-term follow-up | |
| **Age** | Calculated based on the date of admission and date of birth registered in the Danish Civil Registration System. |  |  |
| **Death** | Date of death is available from the Danish Civil Registration System. |  |  |
| **Migration** | Based on data from the Danish Civil Registration System. |  |  |
| **Hospital arrival time** | Time (date, hour, minute) of hospital arrival. If the patient was transferred from one hospital or one unit/department to another er hospital or unit/department, the admissions/contacts were merged, if there was less than 4 hours between the end of one contact to the beginning of the next. Arrival time was the time of the first hospital contact of the merged contacts. | *Danish Clinical Register of Emergency Surgery*^21^ *(described in manuscript – methods section)* | |
| **Type of surgery** | Defined as “Laparotomy”, “laparoscopy” or “converted from laparoscopy to open surgery”  Only available from 2012-2018 | *Danish Clinical Register of Emergency Surgery* | |
| **Time from onset of symptoms** | Defined as time (date, hour, minute) of symptoms onset or “unknown”  Only available from 2014-2018 | *Danish Clinical Register of Emergency Surgery* | |
| **Smoking status** | Defined as never smoker, previous smoker or current smoker | *Danish Clinical Register of Emergency Surgery* | |
| **Co-morbidity** | Defined as Charlson Comorbidity Index (CCI)^48^ based on diagnosis identified in the Danish National Patient Registry 10 years prior to admission. Grouped as: None (CCI 0), low (CCI 1–2), moderate (CCI 3-4) or high (CCI 5+) | | *The Danish National Patient Registry*^44^  *The Danish National Patient Registry-Psychiatry* |
| **Alcohol- or substance abuse** | Defined as registration in one or more of the following registries 5 year prior to admission   1. The National Registry of Alcohol Treatment (available from 2008) 2. The Register of Substance Abusers 3. One or more diagnosis of mental and behavioral disorders due to psychoactive substance use (ICD-10 F10-19) from the Danish National Patient Registry | *The National Registry of Alcohol Treatment* is a national registry established in 2008 that contains data on individuals who have been assigned for treatment of alcoholism. The registry provides statistics on admissions. The municipalities can refer clients to public or private institutions for treating alcoholism. Both are required to report to the Danish Health Authorities.  *The Register of Substance Abusers in Treatment* contains enrolment statistic on client's enrolled in treatment for substance abuse since 1996.  *The Danish National Patient Registry and The Danish National Patient Registry -Psychiatry* (described above) | |
| **Co-habitation** | Defined as living alone or with someone else based on data from Statistics Denmark. Data was available for the status of co-habitation at December 31^st^ each year.  Data from the year of admission was used. If not available (e.g., death at the year of admission), data from the previous year was used. | *Statistics Denmark* is an extensive collection of administrative registers, containing individual-level data from governmental agencies. *The statistics on households and families* (FAM/FAIK) describe the total population living in Denmark. Daily deliveries from the Central Population Register (CPR) provide the basis for the statistics. Statistics are produced covering three different kinds of units: households, families and persons. The statistics describe these units based on household- and family-related variables (e.g. type of family, size of family, type of household). The basis for the statistics consists solely of CPR data on sex, age, marital status, references to spouses and parents, and address specification. The address data form the basis of the division into households, municipalities and regions. *The personal income statistics* (IND) only describes income for persons who are at least 15 years old at the end of the year and who are fully liable to pay tax in the year concerned. The a-income statistics mainly comprise of wages and transfers. The a-income amounts to 90 per cent of the total gross income. *The employment registry* RAS is an annually labor market statistic based on the population’s connection to the labor market on the last working day in November. *The Educational Attainment Register* (UDFF) gather information about the highest completed education for each single person based on the information in The Student Register and The Qualification Register. The primary data source to these statistics is the Student Register with data from 1974 onwards. | |
| **Income** | **Household income** was for the entire cohort (including children) using a definition from Statistics Denmark based on an algorithm including number of adults and children in the household. Household income was grouped as “above national median”, “below national median” or “poverty” (defined as less than 50% of national median).  If income was not available the year of admission (e.g. death), the income of the previous year was used. |  |  |
| **Adherence to workforce** | **Household level** (best in household) for the entire cohort at the year of the admission. Available from 2008 and onwards.  Adherence to workforce was grouped as: Working (incl. fulltime education), age-retirement or social subsidy (public benefits).  If not available at the year of admission due to e.g. death, data from the previous year was used. |  |  |
| **Education** | Defined according to ISCED and the European consensus definitions as Low, Middle or High.^49^ |  |  |

**Supplementary table 4: Proportions with (95%CI) or median with [IQR] for process and outcome measures from the Danish Clinical Register of Emergency Surgery according to mental health history.**

|  | **No history of mental illness** | **History of minor mental illness** | **History of moderate mental illness** | **History of major mental illness** | ***Missing**** |
| --- | --- | --- | --- | --- | --- |
| **Surgical process measures** | | | | |  |
| **3**  **Time-to-antibiotics**, *median* | 208 min [86;419] | 182 min [104;384] | 242 min [104;613] | 314 min  [109;543] | *324 of 1.134 patients 2014-2018* |
| **4**  **Pre-operative risk stratification** | 33%  [30;37] | 40%  [32;47] | 24%  [12;39] | 33%  [19;49] | *168 of 1.134 patients 2014-2018* |
| **5**  **Pre-operative optimization** | 76%  [72;79] | 80%  [73;85] | 69%  [53;82] | 92%  [79;98] | *230 of 1.134 patients 2014-2018* |
| **6**  **Time-to-surgery,** *median* | 5.8 hours [3.5;10.3] | 6.1 hours [3.6;11.0] | 6.4 hours [3.8;10.3] | 6.5 hours  [4.0;13.7] | *84 of 4.767 patients 2004-2018* |
| **Outcome measures** | | | | |  |
| **7**  **30-day mortality** | 20% [19;22] | 30% [28;33] | 16% [11;22] | 27% [21;34] | *0* |
| **8**  **Days alive and out-of-hospital at 90-days follow-up,** *median* | 71 days [0;83] | 50 days [0;80] | 70 days [5;83] | 55 days [0;80] | *0* |

*according to period available – please see table 1 in the main manuscript

**Supplementary table 5: Changes in 30-days mortality and days-alive-and-out-of-hospital in the study period 2004-2018**

| **Years** | **2004-2005** | **2006-2007** | **2008-2009** | **2010-2011** | **2012-2013** | **2014-2015** | **2016-2018** |
| --- | --- | --- | --- | --- | --- | --- | --- |
| ***Number of patients*** | *n=777* | *n=767* | *n=708* | *n=718* | *n=663* | *n=494* | *n=640* |
| **Mental illness** proportion none/minor/  moderate/major | 61%  32%  3%  4% | 61%  30%  5%  4% | 65%  27%  4%  4% | 66%  25%  4%  5% | 71%  21%  4%  4% | 71%  20%  5%  4% | 74%  18%  4%  4% |
| **30 days mortality** proportion and 95%-CI | 27.9% (24.8;31.2) | 24.5% (21.5;27.7) | 24.9% (21.7;28.2) | 27.2% (23.9;30.6) | 18.1% (15.2;21.2) | 17.6% (14.4;21.3) | 17.2% (14.3;20.3) |
| **Days-alive-and-out-of-hospital 90-days follow-up,**  median and IQR | 61 days [0;82] | 63 days [0;82] | 62 days [0;82] | 53 days [0;82] | 65 days [3;82] | 78 days [32;84] | 77 days [10;84] |

**Supplementary table 6: Differences in medians and 75%-quartiles and concordance index**

|  |  |  | **No history** | **History of minor mental illness** | **History of moderate mental illness** | **History of major mental illness** |
| --- | --- | --- | --- | --- | --- | --- |
| **Time-to-antibiotics**  TIME DIFFERENCES | **Differences median**  (minutes) 95% CI | Unadjusted  Sex/age  Sex/age/CCI | REF  REF  REF | -23 [-63;17]  -42 [-86;2]  -34 [-80;13] | 35 [-67;136]  20 [-74;114]  29 [-65;123] | 83 [-78;243]  78 [-88;243]  80 [-90;250] |
|  | **Differences 75%-quartile**  (minutes) 95% CI | Unadjusted  Sex/age  Sex/age/CCI | REF  REF  REF | -42 [-125;43]  -68 [-177;40]  -50 [-147;47] | 188 [-295;670]  73 [-431;578]  139 [-337;615] | 105 [-156;368]  96 [-176;368]  100 [-173;372] |
|  | **Concordance index** 95% CI | Unadjusted  Sex/age/CCI | REF  REF | 0.51 [0.49;0.56]  0.51 [0.46;0.56] | 0.53 [0.43;0.63]  0.47 [0.37;0.57] | 0.56 [0.46;0.66]  0.56 [0.47;0.66] |
| **Time-to-surgery**  TIME DIFFERENCES | **Differences median**  (minutes) 95% CI | Unadjusted  Sex/age  Sex/age/CCI | REF  REF  REF | 17 [-4;38]  2 [-22;25]  2 [-21;26] | 33 [-8;73]  34 [-11;79]  35 [-10;80] | 41 [-27;109]  32 [-25;90]  36 [-26;98] |
|  | **Differences 75%-quartile**  (minutes) 95% CI | Unadjusted  Sex/age  Sex/age/CCI | REF  REF  REF | 41 [-28;110]  20 [-54;95]  29 [-34;93] | 2 [-167;171]  12 [-135;160]  -10 [-143;123] | 196 [4;388]  234 [25;442]  227 [33;420] |
|  | **Concordance index** 95% CI | Unadjusted  Sex/age/CCI | REF  REF | 0.51 [0.49;0.53]  0.51 [0.49;0.53] | 0.49 [0.45;0.53]  0.49 [0.45;0.53] | 0.55 [0.51;0.59]  0.55 [0.51;0.59] |
